# Efficacy of Dual-Antibiotic-Loaded Bone Cement Against Multi-Drug-Resistant *Staphylococcus aureus* and *Enterococcus faecalis* in a *Galleria mellonella* Model of Periprosthetic Joint Infection

**DOI:** 10.3390/antibiotics14121280

**Published:** 2025-12-17

**Authors:** You Zhao, Gopala Krishna Mannala, Raphaëlle Youf, Martina Humez, Ruth Schewior, Klaus-Dieter Kühn, Volker Alt, Martijn Riool

**Affiliations:** 1 Department of Trauma Surgery, University Hospital Regensburg, Franz-Josef-Strauß-Allee 11, 93053 Regensburg, Germanygopala-krishna.mannala@klinik.uni-regensburg.de (G.K.M.); raphaelle.youf@klinik.uni-regensburg.de (R.Y.); volker.alt@klinik.uni-regensburg.de (V.A.); 2 Department of Orthopaedic Surgery, The People’s Hospital of Hechi, Hechi 547000, China; 3 Institute of Microbiology and Molecular Biology, Justus-Liebig-Universität Giessen, Heinrich-Buff-Ring 26, 35392 Giessen, Germany; martina.humez@mikrobio.med.uni-giessen.de; 4 Heraeus Medical GmbH, Philipp-Reis-Str. 8-13, 61273 Wehrheim, Germany; 5 Department of Orthopaedics and Trauma, Medical University of Graz, Auenbruggerplatz 5, 8036 Graz, Austria

**Keywords:** antibiotic-loaded bone cement (ALBC), polymethylmethacrylate (PMMA), *Galleria mellonella*, prosthetic joint infection (PJI), multidrug-resistant bacteria

## Abstract

**Background:** Antibiotic-loaded bone cement (ALBC) is widely used for local antibiotic delivery in joint arthroplasty to prevent and treat prosthetic joint infections (PJIs). In this study, we evaluated the efficacy of cemented Kirschner (K)-wires coated with various ALBC formulations using a *Galleria mellonella* infection model against multidrug-resistant (MDR) *Staphylococcus aureus* and *Enterococcus faecalis*. **Methods:** We tested commercially available bone cements, including gentamicin-only formulations (PALACOS R+G) and dual-antibiotic formulations, combining gentamicin with either clindamycin (COPAL G+C) or vancomycin (COPAL G+V), alongside an antibiotic-free control (PALACOS R). In vitro assays—including minimum inhibitory/bactericidal concentration (MIC/MBC) determination, antibiotic release kinetics, agar diffusion, and antibiofilm evaluations—demonstrated effective antibiotic release and significant antimicrobial activity against both planktonic and biofilm-associated bacteria. **Results:** In vivo, ALBC-coated K-wires were well tolerated in *G. mellonella* and significantly protected the larvae from *S. aureus* infection compared to controls. Notably, dual-antibiotic formulations provided superior protection, correlating with substantial reductions in bacterial colonisation on implant surfaces and in surrounding tissues. **Conclusions:** These findings support the utility of the *G. mellonella* model as a high-throughput, cost-effective platform for the preclinical evaluation of antimicrobial strategies to prevent and treat PJIs and further demonstrate the effectiveness of dual-loaded ALBC against multidrug-resistant bacteria.

## 1. Introduction

Orthopaedic implants have transformed fracture fixation and joint replacement, greatly improving patient outcomes. In the USA, the annual number of procedures is projected to reach 1.22 million total knee arthroplasties (TKA) and 719,000 total hip arthroplasties (THA) by 2040 [1]. Despite their high success rates, these procedures frequently require revision surgeries, primarily due to aseptic loosening and infection. Prosthetic joint infections (PJIs) account for approximately 15% and 25% of THA and TKA revision surgeries, respectively [2]. A recent report by the European Bone and Joint Infection Society (EBJIS) indicated that, out of approximately two million joint replacements performed in Europe in 2019, over 20,000 resulted in PJIs, imposing a substantial economic burden on healthcare systems [3].

PJIs are predominantly caused by *Staphylococcus aureus*, coagulase-negative staphylococci, *Streptococcus* spp. and *Enterococcus* spp. These pathogens form biofilms on implant surfaces, facilitating bacterial persistence and resistance to antimicrobial therapies [4,5]. Furthermore, recent evidence shows that bacteria can evade host immune defences by colonising implant surfaces and forming biofilms [6]. The involvement of multidrug-resistant (MDR) pathogens in PJIs further complicates management, often requiring multiple cement spacer exchanges, increasing the risk of infection recurrence, and substantially adding to healthcare costs [7].

Cemented implants play a crucial role in managing PJIs, particularly in two-stage revision procedures and cases involving limb shortening, disuse osteopenia, and extensive bone and soft tissue loss [8]. Strategies such as antimicrobial coatings (e.g., silver, antibiotics) and antibiotic-loaded bone cements (ALBCs) have been widely adopted to mitigate these complications [9]. Poly(methyl methacrylate) (PMMA) bone cement, especially formulations loaded with single (e.g., gentamicin, tobramycin) or dual antibiotics (e.g., gentamicin combined with clindamycin or vancomycin), enables localised antibiotic delivery and is integral to two-stage exchange protocols [10]. Clinical studies suggest that dual-antibiotic-loaded formulations yield superior outcomes compared to single-antibiotic alternatives [11], which is supported by in vitro evidence demonstrating enhanced antibiotic release kinetics and biofilm inhibition [12].

Preclinical models are crucial for understanding PJI pathogenesis and developing therapeutic strategies. However, research on animal models of cemented implant infections remains limited. For example, Lin et al. demonstrated the effectiveness of vancomycin-loaded PMMA bone cement against *S. aureus* in a rabbit model [13]. Due to ethical and regulatory constraints, vertebrate models must adhere to the 3R principles (Replacement, Reduction, Refinement) and guidelines such as PREPARE [14,15]. Consequently, invertebrate models, including *Drosophila melanogaster*, *Caenorhabditis elegans*, and *Galleria mellonella*, have emerged as robust alternatives for infection studies and high-throughput antimicrobial screening [16,17]. Compared with the other two models, *G. mellonella* offers several advantages for infection studies, including a body size large enough for device implantation, tolerance to 37 °C, closely matching human body temperature, and a complex innate immune system with haemocytes functionally analogous to mammalian phagocytes. Importantly, a *G. mellonella* as a model for implant-associated infections has been established, incorporating Kirschner (K)-wires and ALBC to evaluate biofilm formation and antimicrobial efficiency [18,19]. Additionally, Büssemaker et al. demonstrated the feasibility of using a *G. mellonella* implant infection model to evaluate silver coatings against bacterial infections [20].

In the current study, we build upon this model to investigate the antimicrobial properties of cemented K-wires against MDR pathogens, specifically *S. aureus* and *Enterococcus faecalis*. Using survival assays and bacterial burden analyses, we aim to assess the therapeutic efficacy of various antibiotic combinations against these challenging infections.

## 2. Results

### 2.1. S. aureus and E. faecalis Exhibit Resistance to Gentamicin and Clindamycin

The *S. aureus* EDCC 5055 and *E. faecalis* EUCC2 strains were susceptible to vancomycin (MIC: 1 and 1–2 µg/mL, respectively) but resistant to gentamicin and clindamycin (Table 1). The *S. aureus* strain had a gentamicin MIC of 4–8 µg/mL and an MBC of 8–16 µg/mL, whereas its clindamycin MIC was 32 µg/mL with an MBC of 64–128 µg/mL. *E. faecalis* exhibited high-level resistance to gentamicin and clindamycin, with MIC and MBC values above 128 µg/mL. Of note, the gentamicin and clindamycin used for MIC/MBC determination were obtained as standard infusion solutions, whereas the vancomycin tested was derived from a pharmaceutical-grade powder similar to formulations used in industrially manufactured PMMA cements.

Despite their resistance to gentamicin and clindamycin, these MDR strains were selected intentionally to assess ALBC formulations containing gentamicin alone or in combination with clindamycin or vancomycin, to evaluate their efficacy against resistant pathogens.

### 2.2. Cemented K-Wires Suppress Bacterial Growth In Vitro

#### 2.2.1. Antibiotic-Loaded Cemented K-Wires Inhibit *S. aureus* and *E. faecalis* Despite Resistance

**Figure 1:**
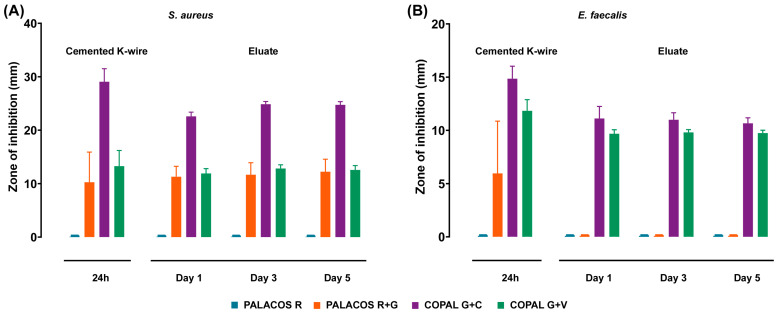
Cemented K-wires inhibit bacterial growth in vitro. Quantification of the mean inhibition zone diameters (in mm) against (**A**) *S. aureus* EDCC 5055 and (**B**) *E. faecalis* EUCC2 using cemented K-wires (*n* = 8 per group). Data are presented as mean ± standard deviation.

The agar diffusion assay showed that antibiotic-loaded cemented K-wires inhibited bacterial growth, despite resistance to gentamicin. Control cemented K-wires (PALACOS R, no antibiotics) did not inhibit *S. aureus* growth, as expected (Figure 1A). However, gentamicin-loaded cemented K-wires (PALACOS R+G) produced an inhibition zone of 10.3 ± 5.6 mm. The gentamicin-vancomycin combination (COPAL G+V) further increased inhibition (13.3 ± 2.9 mm). The gentamicin-clindamycin combination (COPAL G+C) produced the largest inhibition zone (29.1 ± 2.4 mm), which likely reflects its higher gentamicin content (1.0 g per 40 g cement powder) and the resulting increased local antibiotic release compared with PALACOS R+G and COPAL G+V (both containing 0.5 g gentamicin). This effect may additionally be influenced by a potential synergistic interaction between antibiotics with different mechanisms of action.

For *E. faecalis*, PALACOS R did not produce an inhibition zone, as expected, whereas PALACOS R+G generated a small zone (6.0 ± 4.9 mm), suggesting localised activity despite resistance (Figure 1B). COPAL G+V displayed a significant inhibition zone (11.8 ± 1.0 mm), while COPAL G+C showed the highest inhibition (14.9 ± 1.2 mm), indicating superior efficacy.

#### 2.2.2. Antibiotic Eluates Maintain Antimicrobial Activity over Time

To evaluate sustained antimicrobial activity, an agar diffusion assay was performed using eluates from cemented K-wires incubated in PBS. For *S. aureus*, all antibiotic-loaded eluates inhibited bacterial growth after 1 day, with inhibition zones of PALACOS R+G, 11.3 ± 1.9 mm; COPAL G+V, 11.9 ± 0.9 mm; and COPAL G+C, 22.6 ± 0.8 mm (Figure 1A). A slight increase in inhibition zones was observed over 3 and 5 days.

For *E. faecalis*, eluates from COPAL G+V (9.7 ± 0.4 mm) and COPAL G+C (11.1 ± 1.1 mm) produced significant inhibition after 1 day (Figure 1B). No inhibition was detected with PALACOS R or PALACOS R+G, and no further increase was observed at later time points.

### 2.3. Antibiotic Release from Cemented K-Wires Shows a Burst on Day 1 Followed by a Slower Sustained Phase

**Figure 2:**
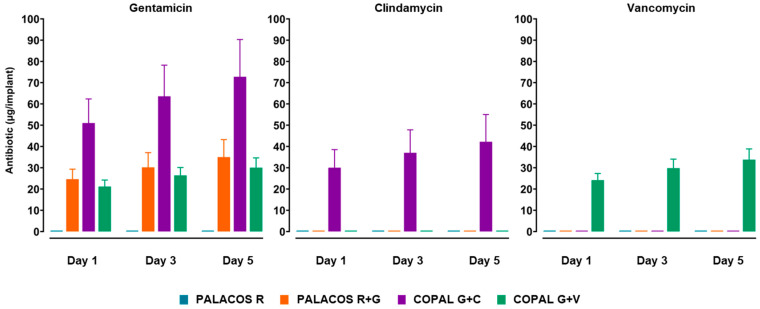
Antibiotic release kinetics from cemented K-wires. Quantitative analysis of cumulative antibiotic release for gentamicin, clindamycin, and vancomycin over five days, as determined by HPLC-MS/MS (*n* = 5 per group). A pronounced burst release was observed on day 1, followed by a slower, sustained phase. Data are presented as mean ± standard deviation.

To evaluate antibiotic release kinetics, cemented K-wires were incubated in PBS, and eluates were analysed on days 1, 3, and 5 using HPLC (Figure 2).

A pronounced burst release was observed on day 1, after which the rate of release declined but continued at a lower, sustained level. PALACOS R+G released 24.6 ± 4.2 µg of gentamicin on day 1, with cumulative amounts increasing to 30.2 ± 6.9 µg on day 3 and 35.0 ± 8.3 µg on day 5 (Figure 2). COPAL G+C showed the highest overall release, with 51.0 ± 10.2 µg of gentamicin and 30.0 ± 7.6 µg of clindamycin on day 1, followed by a gradual increase over time. COPAL G+V exhibited a similar burst-sustained profile for gentamicin and vancomycin. Overall, COPAL G+C released nearly twice as much gentamicin as PALACOS R+G and COPAL G+V, reflecting its higher antibiotic loading.

### 2.4. Cemented K-Wires Exhibit Antibiofilm Activity In Vitro

#### 2.4.1. Dual-Antibiotic-Loaded K-Wires Effectively Disrupt *S. aureus* Biofilms

**Figure 3:**
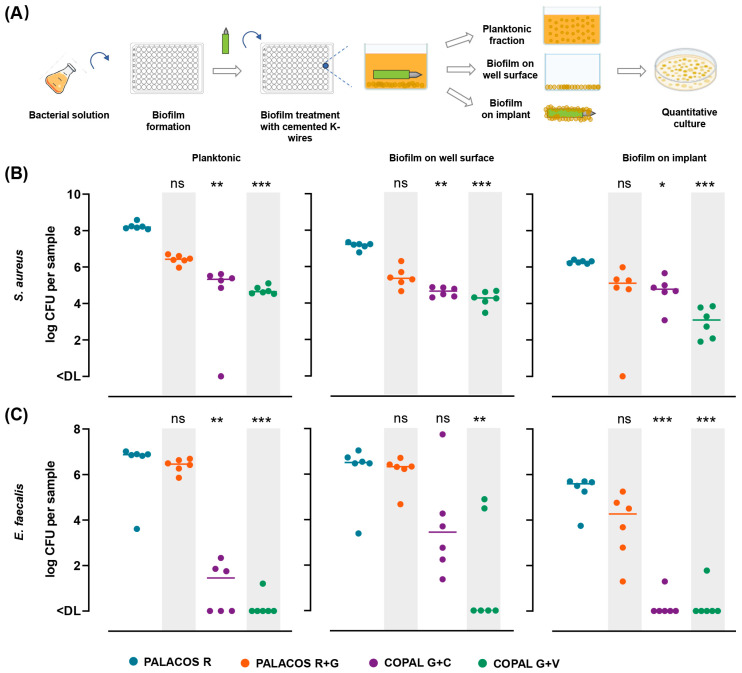
Cemented K-wires exhibit antibiofilm activity in vitro. (**A**) Schematic representation of the biofilm treatment protocol, in which pre-formed *S. aureus* and *E. faecalis* biofilms were treated with cemented K-wires. (**B**) Quantification of viable *S. aureus* bacteria (log CFU) retrieved from the planktonic fraction (left), the biofilm on the well surface (middle), and the cemented K-wire surface (right). (**C**) Quantification of viable *E. faecalis* bacteria (log CFU) in the same compartments as in panel (**B**). Horizontal lines indicate median values (*n* = 6 for each compartment). Data were analysed using the Kruskal–Wallis rank-sum test, with statistical significance denoted as: * = *p* < 0.05, ** = *p* < 0.01, *** = *p* < 0.001), ns = non-significant.

To evaluate the impact of cemented K-wires on pre-formed biofilms, *S. aureus* and *E. faecalis* biofilms were established in 96-well plates, followed by the placement of cemented K-wires (Figure 3A). The antimicrobial efficacy of released antibiotics was assessed by quantifying the bacterial load in three compartments: (i) planktonic bacteria, (ii) biofilm on the well surface, and (iii) bacterial colonisation on the cemented K-wire surface.

For *S. aureus* biofilms, K-wires containing dual-antibiotic-loaded bone cement (COPAL G+C and COPAL G+V) significantly reduced the pre-formed biofilms on the well surfaces, decreased planktonic bacterial counts, and inhibited bacterial colonisation on the implant surface (Figure 3B). Compared to the PALACOS R control, COPAL G+C and COPAL G+V achieved a more than 1.5-log reduction in bacterial burden across all three compartments. In contrast, mono-antibiotic-loaded K-wires (PALACOS R+G) caused a modest 1.2-log reduction on the surface of the implant, indicating partial biofilm inhibition.

#### 2.4.2. Vancomycin-Loaded Cemented K-Wires Reduce *E. faecalis* Biofilms

For *E. faecalis* biofilms, COPAL G+C and COPAL G+V cemented K-wires significantly reduced bacterial burden in the planktonic fraction and bacterial colonisation of the implant surface (at least *p* < 0.01 in all cases) (Figure 3C). The greatest reduction in biofilm on the well surface was observed with COPAL G+V (*p* < 0.01). However, COPAL G+C did not significantly reduce biofilm on the well surface (*p* = 0.135), possibly due to limited efficacy of clindamycin against established *E. faecalis* biofilms. PALACOS R+G showed no significant impact on *E. faecalis* biofilms, planktonic bacteria, or bacterial colonisation of the K-wire surface.

### 2.5. Antibiotic-Loaded Cemented K-Wires Prevent Biofilm Infections In Vivo

#### 2.5.1. Dual-Antibiotic-Loaded Cemented K-Wires Protect Against Biofilm-Associated Pathogenicity

**Figure 4:**
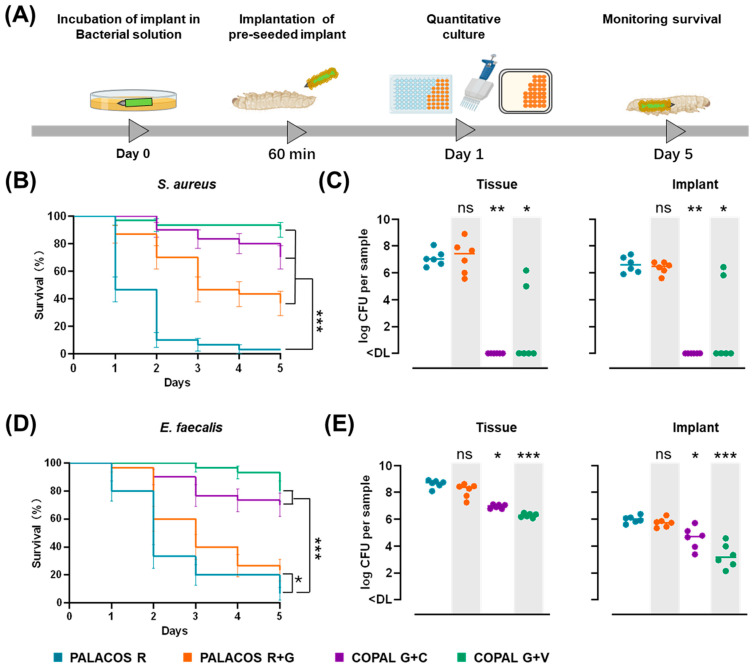
Antibiotic-loaded cemented K-wires prevent biofilm implant infections in *G. mellonella*. (**A**) Schematic representation of the biofilm infection model, in which cemented implants were pre-incubated for 60 min in *S. aureus* or *E. faecalis* suspensions (0.5–1 × 10^7^ CFU/mL) before implantation. The mean number of *S. aureus* adhered to the surface before implantation was log 4.7 CFU (PALACOS R), log 2.8 CFU (PALACOS R+G) and log 3.5 CFU (COPAL G+V) per implant, whereas no bacteria could be observed on COPAL G+C (<DL; *n* = 3 per group). The mean number of *E. faecalis* adhered to the surface before implantation was log 5.9 CFU (PALACOS R), log 5.5 CFU (PALACOS R+G), log 3.9 CFU (COPAL G+C) and log 1.3 CFU (COPAL G+V) per implant (*n* = 3 per group). (**B**,**D**) Kaplan–Meier curves showing percent survival (±SEM) over 5 days for larvae infected with *S. aureus* (**B**) or *E. faecalis* (**D**) after implantation of pre-incubated cemented K-wires. (**C**,**E**) Bacterial burden (log CFU) in larval tissue and on the cemented K-wire surface after 24 h for *S. aureus* (**C**) and *E. faecalis* (**E**). Horizontal lines represent median values. Data were analysed from three independent experiments (*n* = 10 larvae per experiment) using the log-rank test for survival and the Kruskal–Wallis rank-sum test for bacterial burden. (* = *p* < 0.05, ** = *p* < 0.01, *** = *p* < 0.001, ns = non-significant). The detection limits (DL) were 5 CFU/tissue and 3 CFU/implant.

The ability of cemented K-wires to prevent biofilm formation was evaluated in a *G. mellonella* biofilm infection model (Figure 4A). For *S. aureus* biofilm infections, after 5 days, survival was significantly higher with PALACOS R+G (36.7 ± 8.8%; *p* < 0.001), COPAL G+C (70.0 ± 8.4%; *p* < 0.001), and COPAL G+V (90.0 ± 5.5%; *p* < 0.001), compared to non-loaded controls (PALACOS R; 3.3% survival) (Figure 4B).

A similar finding was observed for *E. faecalis* biofilm infections: PALACOS R+G (23.3 ± 7.7%; *p* < 0.05), COPAL G+C (70.0 ± 8.4%; *p* < 0.001), and COPAL G+V (80.0 ± 7.3%; *p* < 0.001), compared to the control (PALACOS R; 6.7 ± 4.6% survival) (Figure 4D).

#### 2.5.2. Dual-Antibiotic-Loaded Cemented K-Wires Reduce Bacterial Load on Implants and in Tissue

To assess bacterial dissemination from biofilms, bacterial burden was quantified in larval tissue and on implant surfaces 24 h post-implantation.

For *S. aureus*, COPAL G+C and COPAL G+V significantly reduced bacterial burden by at least 6.6-log in both implant and tissue samples (*p* < 0.01 and *p* < 0.05, respectively), leading to bacterial clearance in nearly all samples (Figure 4C). PALACOS R+G showed no substantial reduction (log 7.4 CFU/tissue, log 6.5 CFU/implant), confirming limited efficacy against biofilms. The control group (PALACOS R) exhibited log 7.0 CFU/tissue and log 6.6 CFU/implant, confirming extensive bacterial colonisation.

For *E. faecalis*, COPAL G+V significantly reduced bacterial counts by over 2.4-log on implants and in tissue (*p* < 0.001 for both; Figure 4E), whereas COPAL G+C resulted in a smaller yet statistically significant ≥1.3-log reduction (*p* < 0.05 for both) compared to the control (PALACOS R: log 8.7 CFU/tissue, log 6.0 CFU/implant). PALACOS R+G did not significantly affect bacterial burden (log 8.4 CFU/tissue, log 5.8 CFU/implant).

These results demonstrate that dual-antibiotic-loaded cemented K-wires offer superior protection against biofilm infections by significantly reducing bacterial load and preventing systemic infection.

### 2.6. Antibiotic-Loaded K-Wires Prevent Haematogenous Implant Infection in G. mellonella

#### 2.6.1. Antibiotic-Loaded Cemented K-Wires Increase Survival in *S. aureus* and *E. faecalis* Infected Larvae

**Figure 5:**
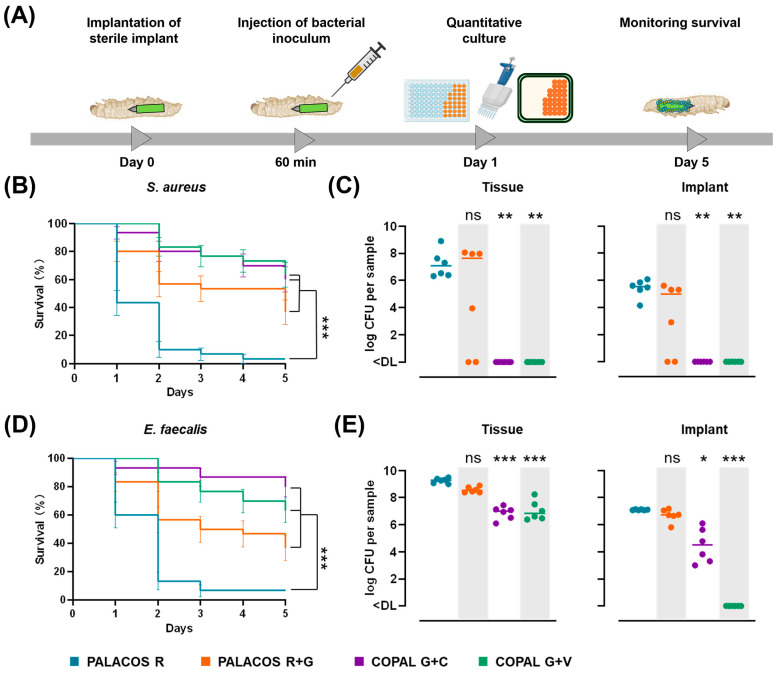
Antibiotic-loaded cemented K-wires prevent haematogenous implant infection in *G. mellonella*. (**A**) Schematic representation of the haematogenous infection model, where larvae received cemented K-wire implantation 60 min prior to infection with *S. aureus* or *E. faecalis* (5 × 10^4^ CFU/larva). (**B**,**D**) Kaplan–Meier survival curves showing the percent survival (±SEM) over 5 days for larvae infected with *S. aureus* (**B**) or *E. faecalis* (**D**) after implantation of cemented K-wires loaded with gentamicin alone (PALACOS R+G) or in combination with clindamycin (COPAL G+C) or vancomycin (COPAL G+V). Non-loaded implants (PALACOS R) served as controls. (**C**,**E**) Bacterial burden (log CFU) in larval tissue and on the cemented implant K-wire surface after 24 h of incubation for *S. aureus* (**C**) and *E. faecalis* (**E**). Horizontal lines represent median values. Data were analysed from three independent experiments (*n* = 10 larvae per experiment) using the log-rank test for survival and the Kruskal–Wallis rank-sum test for bacterial burden. (* = *p* < 0.05, ** = *p* < 0.01, *** = *p* < 0.001, ns = non-significant). The detection limits (DL) were 5 CFU/tissue and 3 CFU/implant.

The in vivo antimicrobial efficacy of cemented K-wires was investigated using a *G. mellonella* haematogenous infection model, in which antibiotic-loaded K-wires were implanted before infection with *S. aureus* or *E. faecalis* (Figure 5A).

For *S. aureus* infections, larvae implanted with antibiotic-loaded cemented K-wires demonstrated significantly improved survival rates compared to the non-loaded control (PALACOS R, 3.3% survival): COPAL G+V (63.3 ± 8.8%; *p* < 0.001), COPAL G+C (60.0 ± 8.9%; *p* < 0.001) and PALACOS R+G (36.7 ± 8.8%; *p* < 0.001) (Figure 5B).

For *E. faecalis* infections, antibiotic-loaded cemented K-wires also significantly improved survival, with COPAL G+V showing the highest protection (80.0 ± 7.3%; *p* < 0.001), followed by COPAL G+C (63.3 ± 8.8%; *p* < 0.001) and PALACOS R+G (36.7 ± 8.8%; *p* < 0.001), compared to PALACOS R (6.7% survival; Figure 5D).

**Figure A1:**
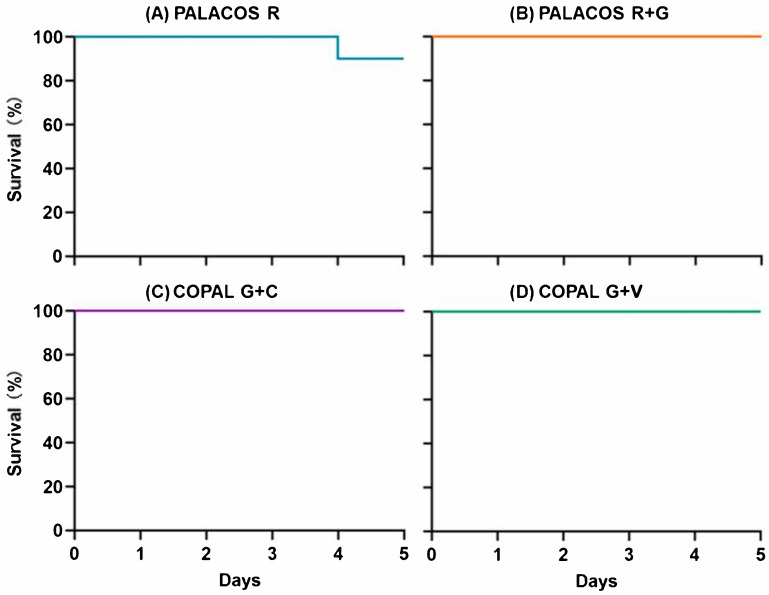
Biocompatibility assay of cemented K-wires in *G. mellonella*. Kaplan–Meier survival curves showing the percentage of larvae surviving over 5 days following implantation with cemented K-wires composed of: (**A**) non-loaded control bone cement (PALACOS R), (**B**) ALBC containing gentamicin alone (PALACOS R+G), (**C**) dual-antibiotic bone cement with gentamicin and clindamycin (COPAL G+C), and (**D**) dual-antibiotic bone cement with gentamicin and vancomycin (COPAL G+V). No significant differences in survival were observed among the groups (*n* = 10 larvae per group).

Notably, PALACOS R+G significantly increased survival in larvae infected with *S. aureus* and *E. faecalis*, despite resistance to gentamicin, suggesting that high local antibiotic concentrations may overcome resistance locally and hinder infection progression. Importantly, sterile cemented implants had no impact on larval survival, confirming that implantation alone did not affect viability (Figure A1).

#### 2.6.2. Dual-Antibiotic-Loaded Cemented K-Wires Eliminate Bacteria from Implants and Surrounding Tissue

To further evaluate the antimicrobial efficacy, bacterial burden was quantified in larval tissue and on the cemented K-wire surface after 24 h. For *S. aureus* infections, COPAL G+C and COPAL G+V reduced bacterial counts in tissue by approximately 7.1-log and on the implant surface by 5.5-log (*p* < 0.01 in all cases), leading to complete bacterial clearance in all cases (Figure 5C). In contrast, PALACOS R+G did not significantly reduce bacterial burden (log 7.6 CFU/tissue, log 5.0 CFU/implant) compared to the control (PALACOS R; log 7.1 CFU/tissue, log 5.5 CFU/implant).

For *E. faecalis* infections, COPAL G+C reduced bacterial counts by more than 2.3-log in tissue (*p* < 0.001) and on the implant surface (*p* < 0.05), while COPAL G+V led to a more than 2.4-log reduction in tissue (*p* < 0.001) and to elimination on the implant surface (*p* < 0.001) (Figure 5E). PALACOS R+G failed to significantly reduce bacterial counts, with similar bacterial loads (log 8.5 CFU/tissue, log 6.7 CFU/implant) compared to the control (PALACOS R; log 9.3 CFU/tissue, log 7.1 CFU/implant).

These findings highlight the effectiveness of dual-antibiotic-loaded cemented K-wires in preventing haematogenous *S. aureus* and *E. faecalis* infections, improving survival, and significantly reducing bacterial colonisation on implant surfaces and adjacent tissues.

## 3. Discussion

Commercially available PMMA bone cements play a crucial role in prosthesis fixation, fracture management, bone tumour treatment, and revision surgeries. Their primary functions include securing artificial joints, delivering local antibiotics, and enhancing mechanical stability. In this study, we evaluated the efficacy of antibiotic-loaded cemented implants against MDR *S. aureus* and *E. faecalis* using previously developed *G. mellonella* infection models [19] to mimic PJI. In vitro and in vivo analyses demonstrated that dual-antibiotic-loaded bone cements (COPAL G+C and COPAL G+V) were significantly more effective in biofilm elimination and infection prevention than PALACOS R+G, which contained only gentamicin. These findings are consistent with clinical studies reporting improved infection control with dual-antibiotic ALBC formulations [22,23]. By utilising clinically relevant, commercially available bone cements, we effectively mimicked real-world conditions and strengthened the relevance of the model.

Cemented implants, including the cemented K-wires used in this study, typically exhibit a pronounced burst release of antibiotics during the initial days, followed by a slower, sustained elution phase. Our efficacy assessments therefore primarily reflect this early burst-release period, and the infection control observed in our model is most appropriately interpreted in the context of high local antibiotic concentrations during the acute phase. Although sustained low-level antibiotic release from PMMA can contribute for extended periods [24], its contribution to long-term infection prevention was not directly evaluated here and remains to be further characterised, particularly in relation to late-onset infections.

However, such elevated antibiotic levels also raise concerns about local cytotoxicity. In vitro studies have shown that while gentamicin- or vancomycin-loaded PMMA cements exhibit only mild cytotoxic effects, combinations such as gentamicin + clindamycin can markedly impair cell viability and osteogenic activity [25]. Moreover, the exothermic polymerisation of PMMA generates free radicals that may induce local oxidative stress and tissue damage [26]. In our in vivo compatibility assay (Figure A1), the antibiotic dose in the cemented K-wires caused no observable harm to *G. mellonella* larvae, but caution is warranted when extrapolating these findings to mammalian systems. Promisingly, formulation strategies are being developed to mitigate toxicity without compromising antimicrobial efficacy. For example, co-loading vancomycin with the antioxidant N-acetylcysteine (NAC) has been shown to enhance the antibiotic elution profile and substantially reduce cytotoxicity, maintaining antibacterial activity for more than 35 days at lower vancomycin doses [26]. These findings emphasise the importance of balancing high local antimicrobial potency with host tissue compatibility in the design of ALBC formulations.

Interestingly, PALACOS R+G (containing 0.5 g gentamicin) demonstrated measurable antimicrobial activity against gentamicin-resistant *S. aureus* in the agar diffusion assay. Moreover, COPAL G+C (containing 1 g gentamicin and 1 g clindamycin) exhibited pronounced antimicrobial and anti-biofilm activity against *S. aureus* and *E. faecalis* strains classified as resistant to both gentamicin and clindamycin. Our findings suggest that both PALACOS R+G and COPAL G+C achieve high local antibiotic release, with dual-antibiotic-loaded bone cements providing a higher total amount of released antibiotic, potentially contributing to concentration-dependent bacterial killing and/or synergistic interactions between antibiotics [22,27]. However, true pharmacodynamic synergy was not formally assessed in this study and therefore cannot be concluded from our data. These findings support the view that antibiotics deemed ineffective systemically due to resistance may still exert local antimicrobial effects at high concentrations. Similarly, Metsemaker et al. reported that a doxycycline-coated titanium intramedullary nail provided complete protection against osteomyelitis caused by a doxycycline-resistant *S. aureus* strain, further supporting the importance of high local antibiotic concentrations in infection control [28].

Bacterial biofilms play a crucial role in antibiotic resistance, and our study demonstrates that COPAL G+V exhibited the highest biofilm disruption activity against both bacterial species, aligning with clinical reports of its growing use in PJI treatment [29]. The varying antimicrobial effects observed over time across different ALBC implants, as demonstrated in agar diffusion assays, are likely attributed to differences in antibiotic loading: COPAL G+C (1 + 1 g), COPAL G+V (0.5 + 2 g), and PALACOS R+G (0.5 g). For future in vivo studies, equalising antibiotic concentrations could allow for direct comparison of additive effects between antibiotics.

Notably, our study successfully adapted the *G. mellonella* model for evaluating cemented implants, an important step considering the widespread clinical use of cemented fixation. This model offers a cost-effective, high-throughput approach for investigating PJI prevention and treatment. Our findings, consistent with clinical observations and previous in vivo studies [13] and clinical settings [30], highlight that dual-antibiotic-loaded ALBC (COPAL G+C and COPAL G+V) is significantly more effective than mono-antibiotic ALBC (PALACOS R+G) during the early phase of infection. This is supported by reduced bacterial burden and early biofilm formation at 24 h post-implantation, as well as significantly improved larval survival over the 5-day observation period in both haematogenous and biofilm-associated infection models with MDR *S. aureus* and *E. faecalis*. Nevertheless, we acknowledge that the absence of bacterial quantification at later post-infection stages represents a limitation and may lead to a partial overestimation of efficacy.

A key advantage of the *G. mellonella* model is its low cost, high-throughput capacity, exemption from ethical approval requirements, and its ability to provide preliminary insights that often align with clinical trends [31]. As such, it serves as a preclinical screening tool that helps reduce reliance on vertebrate models and thereby minimises overall animal use. However, important limitations exist: *G. mellonella* lacks adaptive immunity, including antibody production, which plays a critical role in human PJI responses [32,33]. Moreover, the larvae do not possess a musculoskeletal system or bone-implant interface, limiting their ability to model orthopaedic implant environments. Therefore, while the model is valuable for rapid, cost-effective preliminary assessment of antimicrobial efficacy, results must be validated in more advanced systems, such as vertebrate models, before any clinical translation.

Nevertheless, qualitative parallels can be drawn between our findings and results from established vertebrate models of bone and implant-associated infection reported in the literature. Several studies using rat or rabbit models have demonstrated that antibiotic-loaded PMMA cements, particularly those containing vancomycin or broad-spectrum or dual-antibiotic formulations, can significantly reduce *S. aureus* infection, bacterial burden, and inflammatory responses in vivo, while providing sustained local antibiotic release around the implant [34,35,36]. Comparative investigations have also shown that differences in cement formulation and antibiotic composition lead to distinct biological and antimicrobial outcomes, underscoring the critical role of antibiotic-loaded PMMA in infection control [37]. These observations from vertebrate models are consistent with our results, in which the dual-antibiotic-loaded cements COPAL G+V and COPAL G+C outperformed the mono-antibiotic formulation PALACOS R+G in controlling implant-associated infection in the *G. mellonella* model, providing useful biological context for the superior early anti-infective performance of dual-antibiotic PMMA formulations.

Despite these limitations, the *G. mellonella* model remains a valuable tool for investigating implant-associated infections caused by MDR bacterial strains, particularly those belonging to the ESKAPE group (*Enterococcus faecium*, *S. aureus*, *Klebsiella pneumoniae*, *Acinetobacter baumannii*, *Pseudomonas aeruginosa*, and *Enterobacter* species) [38]. These pathogens pose a global health threat due to their ability to evade standard antibiotic treatments and cause persistent implant-associated infections. Future studies could utilise *G. mellonella* infection models to assess the efficacy of ALBC implants with diverse antibiotic combinations against ESKAPE pathogens and polymicrobial infections.

## 4. Materials and Methods

### 4.1. Bacterial Cultures

The MDR bacterial strains *S. aureus* EDCC 5055 and *E. faecalis* EUCC2 were used for all experiments. The *S. aureus* EDCC 5055 strain, characterised by its biofilm-forming capacity, was originally isolated from a wound infection [39]. The *E. faecalis* EUCC2 strain was isolated from a fracture-related infection at University Hospital Regensburg, Regensburg, Germany.

Prior to each experiment, bacteria were revived from frozen stocks (−80 °C) and grown overnight at 37 °C on LB agar plates (Carl Roth, Karlsruhe, Germany). A single colony was used to inoculate an overnight culture in tryptic soy broth (TSB; Merck, Darmstadt, Germany), incubated at 37 °C with shaking at 180 rpm. The overnight culture was diluted 1:100 in fresh TSB and incubated under the same conditions until it reached the mid-logarithmic growth phase. The bacteria were pelleted by centrifugation, washed once with phosphate-buffered saline (PBS; 140 mM NaCl, pH 7.4; Gibco, Life technologies, Paisley, UK), and resuspended in PBS. The bacterial concentration was adjusted for in vitro and in vivo experiments based on optical density measurements at 600 nm.

The inoculum suspension’s final concentration was confirmed through quantitative culture. Briefly, 10-fold serial dilutions of the bacterial suspension were prepared in PBS and duplicate 5 µL aliquots were plated onto LB agar. Colony-forming units (CFU) were enumerated after overnight incubation at 37 °C, and CFU/mL was calculated.

### 4.2. Antibiotics and Bone Cement Formulations

A selection of antibiotics from different classes was used in this study, including the protein synthesis inhibitors gentamicin (aminoglycoside; infusion solution; 50 mg/mL; Sigma-Aldrich Chemie GmbH, Taufkirchen, Germany) and clindamycin (lincosamide; infusion solution; 50 mg/mL; 1A Pharma, Holzkirchen, Germany), as well as vancomycin (glycopeptide; pharmaceutical-grade powder, 1000 mg per vial; Dr. Friedrich Eberth Arzneimittel GmbH, Ursensollen, Germany), which inhibits bacterial cell wall synthesis. Vancomycin powder was reconstituted according to the manufacturer’s instructions to obtain a stock solution of 50 mg/mL, matching the nominal concentration of the gentamicin and clindamycin infusion solutions. Working solutions of all antibiotics were subsequently prepared in Milli-Q water at 2.56 mg/mL and stored at 4 °C, protected from light, until use.

Bone cements used in this study were obtained from Heraeus Medical GmbH (Wehrheim, Germany) and included the following formulations: PALACOS R, an unloaded bone cement containing no antibiotics; PALACOS R+G, a bone cement containing 0.5 g gentamicin; COPAL G+C, a bone cement containing 1 g gentamicin and 1 g clindamycin; and COPAL G+V, a bone cement containing 0.5 g gentamicin and 2 g vancomycin. These bone cements were prepared and analysed as described in subsequent sections.

### 4.3. Antimicrobial Activity of Antibiotics in Solution (MIC/MBC)

To determine the antimicrobial activity of antibiotics, the minimum inhibitory concentration (MIC) and minimum bactericidal concentration (MBC) were assessed according to EUCAST guidelines. For each bacterial strain, a 10 µL aliquot of the inoculum suspension (1 × 10^7^ CFU/mL, resulting in a final concentration of 1 × 10^6^ CFU/mL) was added to 90 µL of two-fold serially diluted antibiotic solutions in TSB (final concentrations ranging from 128 to 0.125 µg/mL) in a 96-well polystyrene flat-bottom microtiter plate (Sarstedt AG, Nümbrecht, Germany). A non-treated control was included, in which bacteria were incubated in TSB without antibiotics. Plates were incubated overnight at 37 °C and 180 rpm in a humidified environment.

After incubation, the wells were visually inspected for bacterial growth to determine the MIC, defined as the lowest antibiotic concentration that completely inhibited visible growth. To determine the MBC, 5 µL aliquots from each well were plated onto LB agar plates to quantify the number of viable bacteria. After overnight incubation at 37 °C, the MBC was identified as the lowest antibiotic concentration that killed ≥99.9% of bacteria within 24 h. Experiments were performed in duplicate, with *n* = 3 for all conditions, and antimicrobial susceptibility was interpreted using EUCAST clinical breakpoints.

### 4.4. Preparation of Cemented Implants

The radiopaque polymer powder (40–43 g, depending on the type of bone cement) was rapidly and thoroughly mixed with 20 mL of monomer liquid in a mixing bowl to form a homogeneous paste. Mixing lasted approximately 20 s. The paste was then transferred into Teflon moulds (Karl Lettenbauer, Erlangen, Germany) using a spatula.

**Figure 6:**
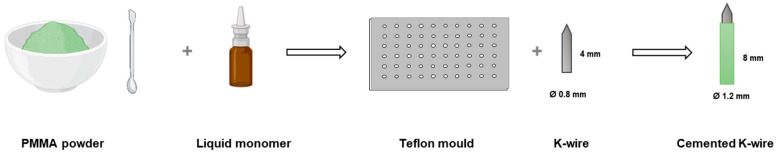
Schematic representation of the sample preparation.

To create cemented implants, 4 mm long K-wire segments with a 0.8 mm diameter (TiAl_6_V_4_; DePuy Synthes, Oberdorf, Switzerland) were rapidly inserted into the bone cement paste within the Teflon mould before the onset of cement polymerization, resulting in implants measuring 8 mm in length and 1.2 mm in diameter (Figure 6). The distal 1.5–2 mm of the K-wire tip was left uncemented to mimic the human situation, in which the articulating part of the prosthesis cannot be cemented. After 30 min, once polymerisation was complete, the samples were carefully removed from the mould using a metal pin. The implants were sharpened with an electric combination tool (Georg Roth GmbH, Fürth, Germany) and sterilised under ultraviolet (UV) light for 30 min before implantation into *G. mellonella* larvae.

### 4.5. Release Kinetics of Antibiotics from Cemented K-Wires

To assess the antibiotic release kinetics from cemented K-wires, samples (*n* = 5 per group) were incubated in 1.2 mL of PBS at 37 °C for up to 5 days. Eluate samples were collected after 1, 3, and 5 days for high-performance liquid chromatography (HPLC) analysis. At each time point, fresh PBS was added to maintain consistent incubation conditions. The concentrations of gentamicin, clindamycin, and vancomycin in the eluates were quantified using HPLC with MS/MS detection. The method was validated according to standard protocols, using one set of matrix calibration standards and two quality control samples. Calibration samples were used to calculate the results, while quality control samples monitored analytical accuracy. Antibiotic release data are presented as cumulative amounts released over time, expressed as mean and standard deviation for all test groups [40].

### 4.6. In Vitro Antimicrobial Activity of Cemented K-Wires

#### 4.6.1. Agar Diffusion Assay

A modified Kirby-Bauer agar diffusion assay [41] was performed to evaluate the inhibition zone of cemented K-wires against *S. aureus* and *E. faecalis*. A bacterial suspension was prepared by suspending five bacterial colonies in 5 mL of TSB. The suspension was evenly spread onto the surface of Mueller Hinton Agar (MHA) II plates, and excess liquid was removed to ensure uniform distribution. After a brief drying period, the cemented K-wires were placed on the inoculated surface, and the plates were incubated for 24 h at 37 °C (*n* = 8 cemented K-wires per group).

The inhibition zones were measured at four positions around each cemented K-wire, and the mean diameter (mm) with standard deviation was calculated. Additionally, cemented K-wires were incubated in 500 µL PBS for up to 5 days. At 1, 3 and 5 days, 5 µL of the eluate was spotted on pre-inoculated MHA plates to assess the antimicrobial activity of the released antibiotics. Plates were incubated for 24 h at 37 °C, and the inhibition zone diameter was measured.

#### 4.6.2. Antibiofilm Assay of Cemented K-Wires

To assess the antibiofilm activity of cemented K-wires (*n* = 6 per group), overnight cultures of *S. aureus* and *E. faecalis* were diluted 1:100 in fresh TSB, and 200 µL of the suspension was added to each well of a 96-well plate (approximately 2 × 10^5^ CFU/well). The plates were incubated at 37 °C and 100 rpm for 24 h to allow biofilm formation.

After incubation, the wells were gently washed with PBS, followed by the addition of 200 µL of fresh TSB and placement of a cemented K-wire in each well. Plates were incubated for another 24 h at 37 °C and 100 rpm. After incubation, three measures of bacterial growth were quantified (Figure 3A): (i) planktonic growth in the medium, (ii) biofilm formation in the well, and (iii) biofilm formation on the implant surface. The medium was collected, and both the implants and wells were rinsed separately with PBS. Subsequently, the implants were then sonicated separately in 500 µL PBS and the wells in 200 µL PBS for 5 min at 45 kHz in a water bath sonicator (Ultrasonic Cleaner USC-T; VWR, Ismaning, Germany) and vortexed for 30 s to detach and disperse adherent biofilm cells. This procedure does not affect bacterial viability [42].

The medium and sonicates were serially diluted ten-fold, and 5 µL droplets of each dilution were plated onto LB agar plates. To enhance the detection limit, an additional 200 µL was directly plated onto LB agar. Plates were incubated overnight at 37 °C, and CFU were enumerated.

### 4.7. G. mellonella Implant Infection Models

#### 4.7.1. Animals

*G. mellonella* larvae were obtained from Evergreen GmbH (Augsburg, Germany) and Fauna Topics GmbH (Marbach am Neckar, Germany) and maintained on wheat germ (Tropic Shop GmbH, Nordhorn, Germany) at room temperature prior to experiments and were incubated at 37 °C following implantation. For each survival experiment, 10 larvae in the last instar stage (weighing approximately 450–550 mg) were used per group, and each experiment was performed in triplicate (*n* = 30 larvae per group). Larvae randomly allocated to treatment or control groups before inoculation. For bacterial quantification on implant surfaces and in larval tissue, an additional set of six larvae per group was used. To assess the in vivo biocompatibility, the larvae received a sterile cemented K-wire, and their survival rate was monitored for 5 days (*n* = 10 larvae per group). All experiments adhered to the ARRIVE guidelines [43].

#### 4.7.2. Biofilm Implant Infection Model

For a biofilm infection, cemented K-wires were pre-incubated with *S. aureus* or *E. faecalis*. The implants were immersed in TSB containing 0.5–1 × 10^7^ CFU/mL of the bacterial inoculum suspension and incubated at 180 rpm for 1 h, following established procedures [19,44]. After incubation, the K-wires were rinsed with PBS and implanted into the posterior segment of each larva by piercing the cuticle with the sharpened end of the implant. The larvae were maintained at 37 °C, and survival was monitored for 5 days. Before implantation, bacterial numbers on additional implants (*n* = 3) were determined using the quantitative culture method described below.

#### 4.7.3. Haematogenous Implant Infection Model

To model haematogenous infection, cemented K-wires were implanted into the posterior segment of the larvae by piercing the cuticle with the sharpened end of the implant. The larvae were then incubated at 37 °C. After 1 h, each larva received an injection of 10 µL of *S. aureus* or *E. faecalis* inoculum suspension (5 × 10^6^ CFU/mL in PBS), corresponding to 5 × 10^4^ CFU/larva, directly at the site of the implanted cemented K-wire. The larvae were maintained at 37 °C, and survival was monitored for 5 days [19,44].

#### 4.7.4. Quantitative Culture

To assess the antimicrobial activity of cemented K-wires, bacterial counts were quantified from the implant surface and larval tissue. At 1-day post-implantation, implants were carefully separated from larval tissue for bacterial quantification. Implants were rinsed with PBS, sonicated in 0.5 mL PBS for 5 min at 45 kHz in a water bath sonicator, and vortexed for 30 s to dislodge adherent bacteria.

Larval tissue samples were homogenised using a Precellys system (VWR). Larvae were surface-disinfected with 70% ethanol and subsequently mechanically disrupted in 1 mL PBS using six large (Ø 2.8–3.2 mm) and ~10 small (Ø 1.4–1.6 mm) yttrium-stabilised zirconium oxide grinding beads (Cerdur, Vechta, Germany). Homogenisation was carried out over six cycles of 30 s at 8000 rpm, with 30 s rest intervals between cycles, under continuous cooling at 4 °C.

The sonicates and homogenates were serially diluted ten-fold, and 5 µL of each dilution was plated onto mannitol salt agar (Sigma-Aldrich) or LB agar plates containing 15 µg/mL gentamicin to suppress the growth of larval bacterial flora. Plates were incubated overnight at 37 °C. To improve detection sensitivity, an additional 200 µL of the undiluted sample was plated. The lower detection limits were 3 CFU per implant and 5 CFU per tissue sample. For visualisation on a logarithmic scale, a value of 1 CFU was assigned when no bacterial growth was detected.

### 4.8. Statistics

Statistical analyses were performed using GraphPad Prism 9.5 (GraphPad Software, San Diego, CA, USA). Bacterial counts were analysed using the Kruskal–Wallis rank-sum test, represented as log_10_ CFU with the median value for each group. Differences in *G. mellonella* survival curves were analysed using the log-rank test. Survival data were represented as mean ± standard error of the mean (SEM) from three independent experiments, each with 10 larvae per group. A *p*-value of <0.05 was considered statistically significant.

## 5. Conclusions

This study shows that dual-antibiotic-loaded cemented implants—combining gentamicin with either clindamycin or vancomycin—effectively reduce bacterial colonisation by MDR *S. aureus* and *E. faecalis* in both haematogenous and biofilm infections. Significant bacterial burden reductions on implant surfaces and surrounding tissues were associated with improved survival outcomes in our infection models. These findings provide strong experimental data supporting the clinical use of dual-antibiotic ALBC formulations as a strategy to reduce revision surgeries and associated morbidity in orthopaedic practice.

## Figures and Tables

**Table 1:** MIC and MBC values of *S. aureus* and *E. faecalis*.

Bacterial Strain	Antibiotic	MIC (µg/mL)	ECOFF ^2^	Interpretation ^1^	MBC (µg/mL)
*S. aureus* EDCC 5055	Gentamicin	4–8	2	R	8–16
	Clindamycin	32	0.25	R	64–128
	Vancomycin	1	2	S	1
*E. faecalis* EUCC2	Gentamicin	>128	128	R	>128
	Clindamycin	>128	-	R	>128
	Vancomycin	1–2	4	S	32

Minimum inhibitory concentration (MIC) and minimum bactericidal concentration (MBC) of antibiotics against *S. aureus* EDCC 5055 and *E. faecalis* EUCC2 (in µg/mL). ^1^ Categorised as susceptible (S) or resistant (R) according to EUCAST, ^2^ including the corresponding epidemiological cut-off (ECOFF) values [21].

## Data Availability

The original contributions presented in the study are included in the article, further inquiries can be directed to the corresponding author.
